# Neural correlates underlying change in state self-esteem

**DOI:** 10.1038/s41598-018-20074-0

**Published:** 2018-01-29

**Authors:** Hiroaki Kawamichi, Sho K. Sugawara, Yuki H. Hamano, Ryo Kitada, Eri Nakagawa, Takanori Kochiyama, Norihiro Sadato

**Affiliations:** 1 Graduate School of Human Health Sciences, Tokyo Metropolitan University, Tokyo, 116-8551 Japan; 2 Division of Cerebral Integration, Department of System Neuroscience, National Institute for Physiological Sciences, Okazaki, 444-8585 Japan; 3 School of Medicine, Faculty of Medicine, Gunma University, Maebashi, 371-8511 Japan; 4 Faculty of Science and Engineering, Waseda University, Tokyo, 169-8555 Japan; 5 Department of Physiological Sciences, The Graduate University for Advanced Studies (Sokendai), Okazaki, 444-8585 Japan; 6 The Japan Society for the Promotion of Science, Tokyo, 102-0083 Japan; 7 School of Social Sciences, Nanyang Technological University, 14 Nanyang Avenue, 637332 Singapore, Singapore; 8 ATR Brain Activity Imaging Center, Sagara-gun, 619-0288 Japan

## Abstract

State self-esteem, the momentary feeling of self-worth, functions as a sociometer involved in maintenance of interpersonal relations. How others’ appraisal is subjectively interpreted to change state self-esteem is unknown, and the neural underpinnings of this process remain to be elucidated. We hypothesized that changes in state self-esteem are represented by the mentalizing network, which is modulated by interactions with regions involved in the subjective interpretation of others’ appraisal. To test this hypothesis, we conducted task-based and resting-state fMRI. Participants were repeatedly presented with their reputations, and then rated their pleasantness and reported their state self-esteem. To evaluate the individual sensitivity of the change in state self-esteem based on pleasantness (i.e., the subjective interpretation of reputation), we calculated evaluation sensitivity as the rate of change in state self-esteem per unit pleasantness. Evaluation sensitivity varied across participants, and was positively correlated with precuneus activity evoked by reputation rating. Resting-state fMRI revealed that evaluation sensitivity was positively correlated with functional connectivity of the precuneus with areas activated by negative reputation, but negatively correlated with areas activated by positive reputation. Thus, the precuneus, as the part of the mentalizing system, serves as a gateway for translating the subjective interpretation of reputation into state self-esteem.

## Introduction

For more than a century, the self has been a central concept in psychological theories^1^. The self is defined as the mental capacity for taking oneself as the object of its own attention^2^. This capacity enables reflected appraisal of self-image, i.e., a person’s conceptualization of themselves, which is an essential component of self-esteem^3^. Because the reflected appraisals require information related to others’ responses obtained through social interaction^4,5^, self-esteem is a product of the social environment. In this sense, self-esteem has a fluid, ever-changing element, known as state self-esteem, in addition to a stable element known as trait self-esteem^6–8^. Trait self-esteem is defined as general self-evaluative feelings over the course of the previous year^7,8^, whereas state self-esteem is defined as current self-evaluative feelings^7,8^.

State self-esteem is highly variable depending on context^9^. Its changeability based on others’ responses during social interactions contributes to maintenance of a positive sense of self-esteem^10^. Furthermore, the state self-esteem acts as “sociometer”^3^, updating the self-esteem through reflected appraisal to monitor the degree to which perceivers are accepted or rejected. Via this function, we form better social relationships by decreasing the possibility of social rejection^3^.

The neural correlates representing state self-esteem were investigated^11^. Eisenberger *et al*. conducted functional magnetic resonance imaging (fMRI) in which participants viewed feedback words (“interesting,” “boring”) from other individuals describing the participant’s previously recorded interview. Participants rated their state self-esteem in response to each feedback word. Eisenberger *et al*. reported greater activity in rejection-related neural regions (dorsal anterior cingulate cortex [dACC], anterior insula [AI]) and mentalizing regions associated with lower state self-esteem. This study was the first to reveal the involvement of the affective pain network and mentalizing network in state self-esteem, particularly in response to negative feedback. Specifically, the cortical midline structures (medial prefrontal cortex [mPFC] and precuneus) of the mentalizing network responsible for inferring mental states of others are involved in self-referential thought^12–16^. In line with previous studies of self-referential thought, the self-representation function in the mPFC^17–19^ and the precuneus^19,20^ also represents reputation from others. Eisenberger *et al*. postulated that state self-esteem is responsive to subjective interpretation of the appraisal of others. However, in their experimental design, personal interpretation of the appraisal of others was not measured. Thus, it remains unknown how the perception of others’ appraisals is translated into state self-esteem.

In this study, we extended Eisenberger’s study by including a measurement of the subjective interpretation (pleasantness) of the appraisal of others, in order to evaluate the effect of this interpretation on the change in state self-esteem. We defined evaluation sensitivity as the rate of change in self-esteem per unit pleasantness induced by reputation information provided during feedback trials. We hypothesized that pleasantness might affect self-esteem to different degrees across individuals, depending on their interpretation of how reputation influences their own self-esteem. Assuming that the change in state self-esteem is correlated with the perceived pleasantness of evaluation words in an individualized manner, we used the slope information from each participant as the evaluation sensitivity, i.e., the rate of change in self-esteem per unit pleasantness. Thus, neural substrates that exhibit evaluation-related activity, which correlates with evaluation sensitivity, should be involved in the process of transforming perception of others’ appraisals into state self-esteem.

We hypothesized that activation of the mPFC or precuneus represents the transformation process leading to change in state self-esteem. Because evaluation sensitivity can be considered as a trait, the corresponding neural substrates can be evaluated by the resting state network without any task load. Here, our expectation was that inter-individual differences in evaluation sensitivity would be represented by functional connectivity with the node involved in transformation of the perception of others’ appraisals into state self-esteem. This node is located in the midline mentalizing structures.

**Figure 1:**
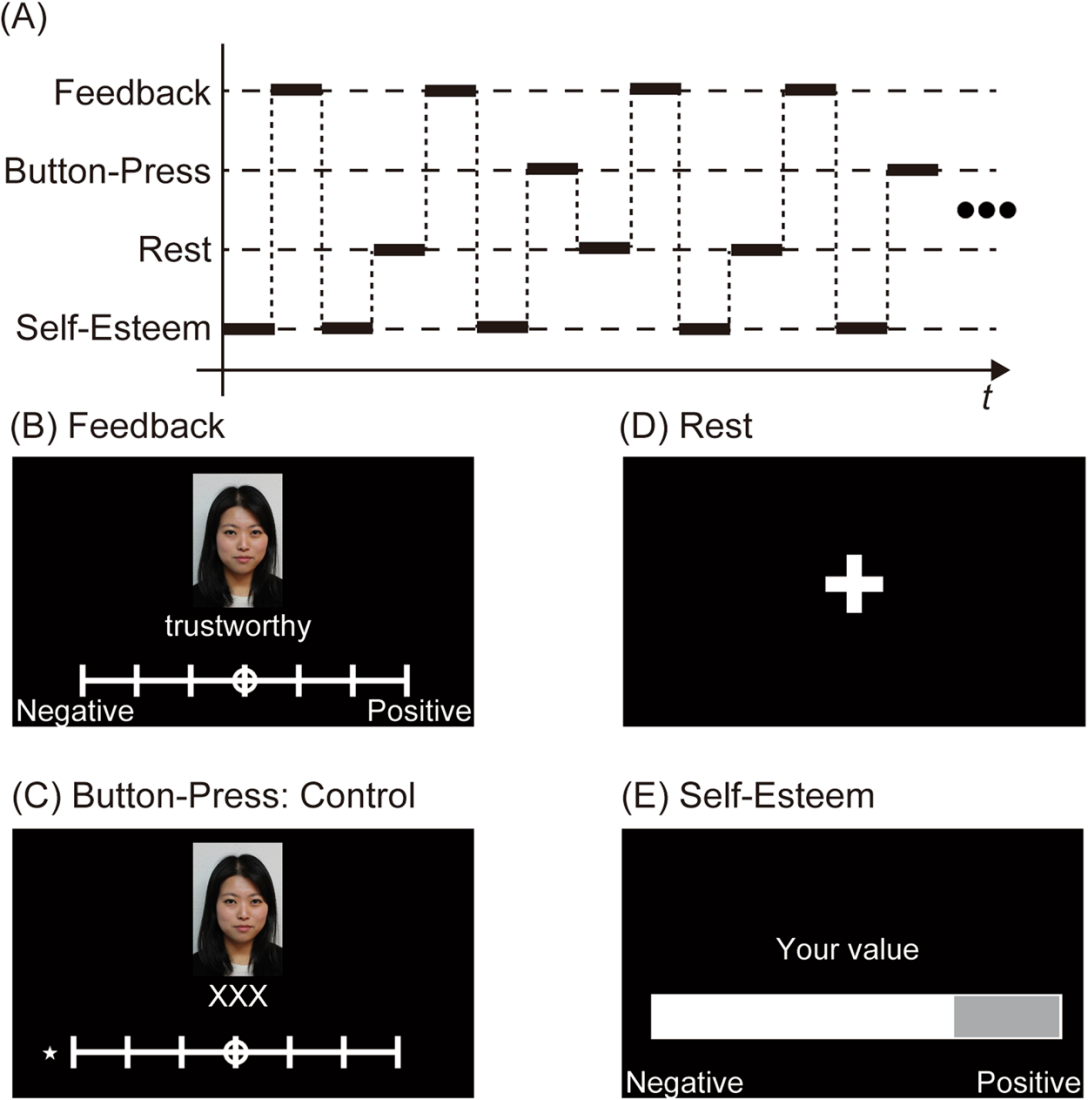
Schematic of the experiment. (**A**) An example condition sequence is shown. The feedback, button-press, and rest trials were presented in a pseudorandom order. (**B**) Visual stimuli in feedback trials showing evaluations. A photograph of the evaluation target (participant) was presented throughout the feedback trials. Adjectives were presented in the lower part of the display. Participants were told that ostensible evaluators selected the adjectives suited for the participants. Participants were required to rate the perceived pleasantness of the presented evaluation. Based on the results of pleasantness ratings, the feedback conditions were categorized as positive, negative, or intermediate. (**C**) Visual stimuli in the button-press trials are shown. Here, ‘XXX’ is displayed instead of adjectives. Participants were required to move the rating toward the side where the star is presented; in this example, participants were required to move the circle to the far left. (**D**) In the rest trial, a fixation cross was presented at the center of the screen for 5 s. (**E**) Visual stimuli of self-esteem trials are shown. Participants were required to rate perceived self-esteem at that time using a visual analog scale ranging from 0 to 100.

In the experiment, each participant viewed evaluation words ostensibly reflecting rating of their behavior by others (reputation) (Fig. 1). After presentation of each evaluation word, the participants provided two ratings: perceived pleasantness of the rating word, and state self-esteem immediately after presentation of the evaluation word. Because the participants of this study were required to rate perceived pleasantness based on evaluation by others regarding a social status (e.g., “trustworthy”), their pleasantness ratings mainly reflected social self-esteem (a subset of self-esteem). They also participated in resting-state fMRI experiment after the task-based fMRI described above.

## Results

### Behavioral Data: Pleasantness Rating and State Self-esteem Rating

The average pleasantness rating ± standard error of the mean (SEM) was 6.29 ± 0.07 for positive evaluation (PE) conditions and 2.61 ± 0.09 for negative evaluation (NE) conditions. Paired *t*-test revealed a significant difference between pleasantness under PE and NE conditions (*t*(27) = 27.64, *p* < 0.001).

The average state self-esteem ± SEM was 67.27 ± 2.10 for PE conditions and 53.38 ± 2.67 for NE conditions. Paired *t*-test revealed a significant difference between state self-esteem under PE and NE conditions (*t*(27) = 6.86, *p* < 0.001).

The average change in state self-esteem ± SEM was 6.76 ± 0.99 for PE conditions and −7.26 ± 1.11 for NE conditions. Paired *t*-test revealed a significant difference between change in state self-esteem under PE and NE conditions (*t*(27) = 6.72, *p* < 0.001). Based on these results, we concluded that state self-esteem is modulated by the valence of received reputation.

**Figure 2:**
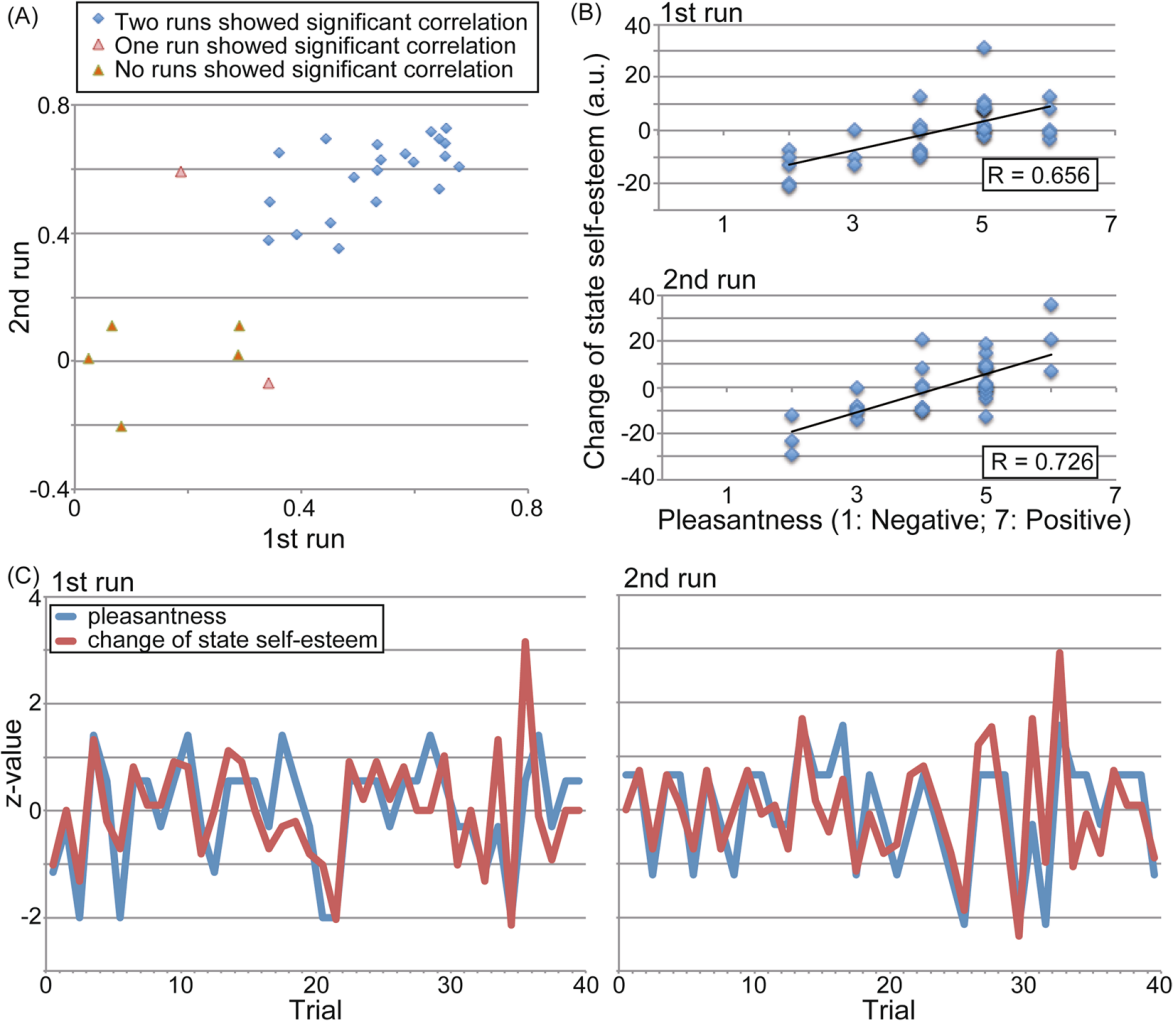
Relationship between the pleasantness rating and change in state self-esteem. (**A**) Correlation coefficients between the pleasantness rating and change in state self-esteem over two runs with 28 participants. The blue square shows data from 21 participants, which exhibited significant correlations over two runs. Light red triangles show data from two participants that exhibited significant correlation over one run. The red triangles show data from five participants that did not exhibit significant correlations. (**B**) Scatter diagram of perceived pleasantness and change in state self-esteem of a typical participant. (**C**) Trial-by-trial standardized value of perceived pleasantness (blue) and change in state self-esteem (red) for a typical participant.

Simple regression analyses between the pleasantness rating and change in self-esteem revealed a significant positive correlation in both runs in 21 out of 28 participants (Fig. 2).

### fMRI Results: Evaluation-Related Activation

**Figure 3:**
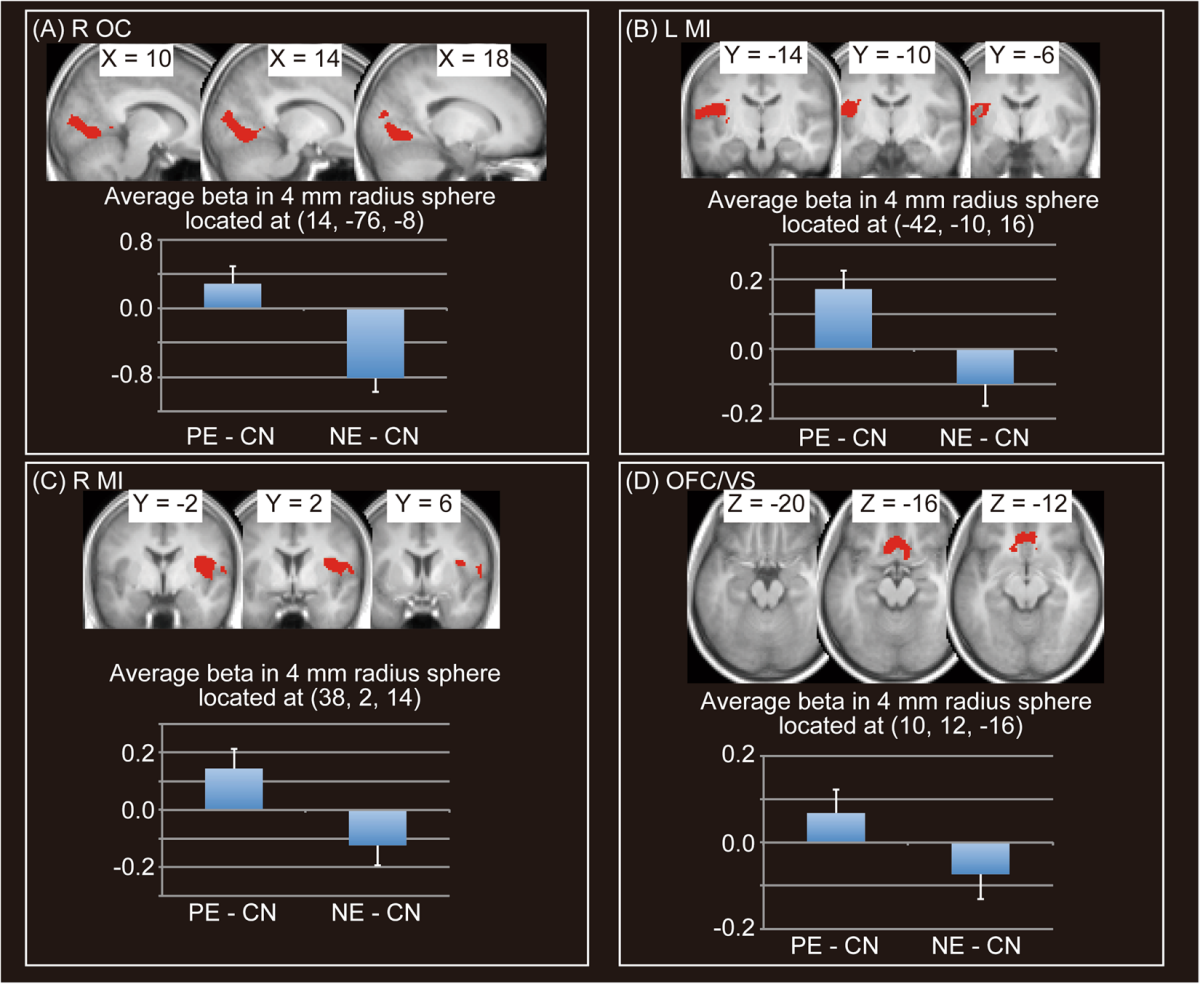
Significant activation for positive evaluation (PE) > negative evaluation (NE). Four significant clusters are shown. We did not show a significant cluster (located at (44, −16, 56)), which showed negative beta value in PE condition. Activation threshold was set at an uncorrected *p* < 0.001 at the voxel level and a family-wise error (FWE) corrected *p* < 0.05 at the cluster level. Peaks of significant clusters in the right occipital cortex (R OC) (**A**), the left middle insula (L MI) (**B**), the R MI (**C**) and orbitofrontal cortex (OFC)/ventral striatum (VS) (**D**), overlaid onto the mean normalized T1 image of 26 participants, are shown in the upper area. Average beta values in spheres of 4-mm radius located at peaks of significant clusters for the two conditions, in comparison with the control condition (positive evaluation [PE] – control [CN], negative evaluation [NE] – CN), are shown in the lower area.

**Figure 4:**
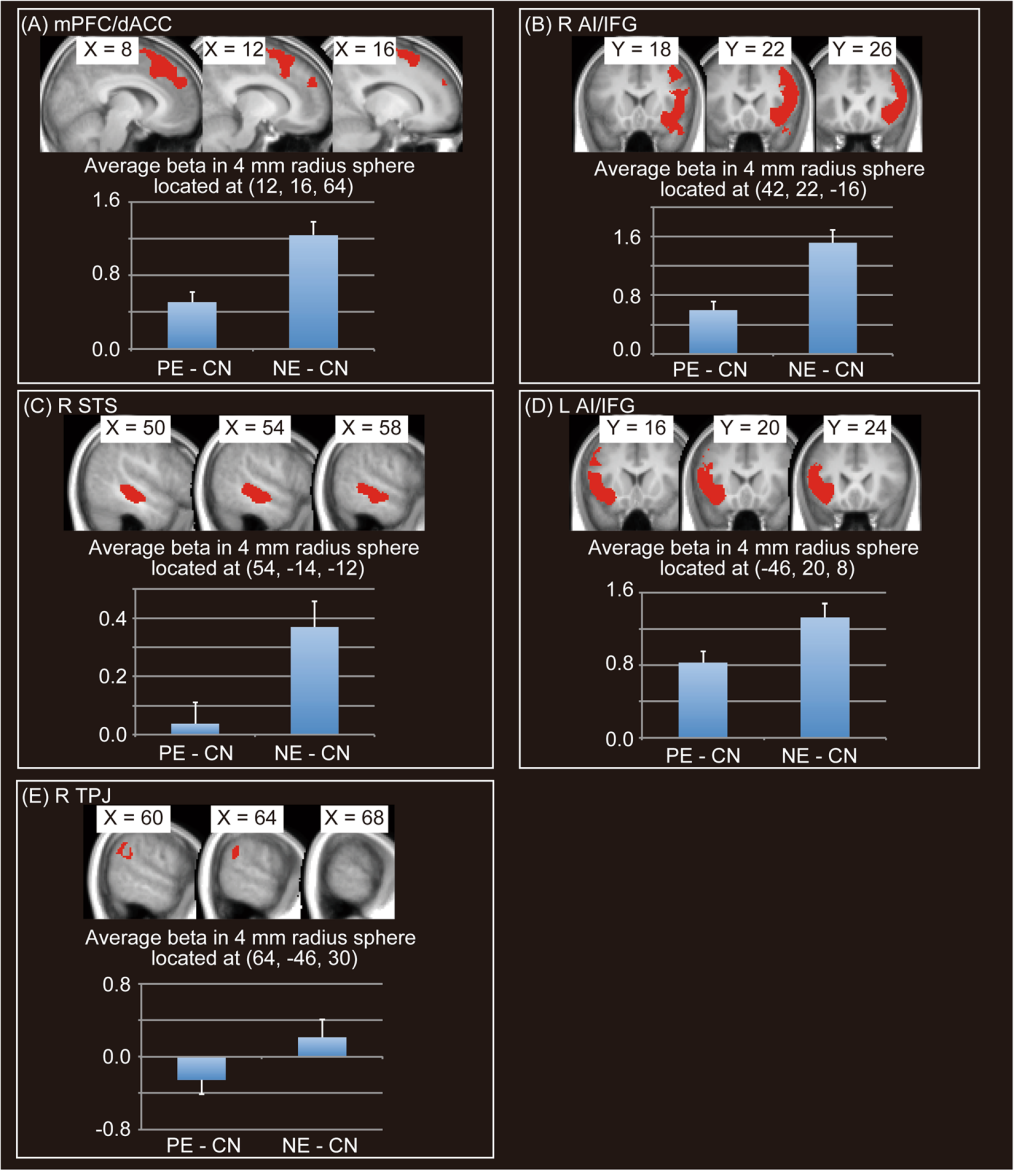
Significant activation for negative evaluation (NE) > positive evaluation (PE). Four significant clusters are shown. Activation threshold was set at an uncorrected *p* < 0.001 at the voxel level and a family-wise error (FWE) corrected *p* < 0.05 at the cluster level. Peaks of significant clusters in medial prefrontal cortex (mPFC)/dorsal anterior cingulate cortex (dACC) (**A**), right anterior insula (R AI)/right inferior frontal gyrus (R IFG) (**B**), right superior temporal sulcus (R STS) (**C**), left AI/IFG (L AI/IFG) (**D**), right temporo-parietal junction (R TPJ) (**E**), overlaid onto the mean normalized T1 image of 26 participants, are shown in the upper area. Average beta values in spheres of 4-mm radius located at peaks of the five significant clusters for the two conditions, in comparison with the control condition (positive evaluation [PE] – control [CN], negative evaluation [NE] – CN), are shown in the lower area.

Positive evaluation conditions were associated with significant activation in regions including the right occipital cortex (OC), middle insula, or orbitofrontal cortex (OFC)/ventral striatum (VS) in comparison with negative evaluation conditions (PE > NE) (Fig. 3 and Table 1). On the other hand, negative evaluation conditions were associated with significant activation in regions including mPFC/dACC, right STS, left AI, right temporoparietal junction (TPJ), or left OC (NE > PE) (Fig. 4 and Table 1).

**Table 1:** Significant activation under positive evaluation (PE) or negative evaluation (NE) conditions.

Cluster *p* (FWE)	Cluster size	x	y	z	*t* value	label
*PE* > *NE*						
<0.001	771	14	−76	−8	9.36	R occipital cortex
		14	−84	2	7.27	R occipital cortex
		2	−90	2	4.09	R occipital cortex
		12	−54	0	3.69	R occipital cortex
		12	−50	2	3.61	R occipital cortex
<0.001	1764	−42	−10	16	8.52	L middle insula
		−46	−24	20	6.87	L parietal operculum
		−58	−20	14	6.63	L parietal operculum
		−44	−40	24	6.37	L supramarginal gyrus
		−46	−36	24	5.87	L supramarginal gyrus
		−46	−20	26	5.69	L parietal operculum
		−38	−2	16	5.58	L middle insula
		−48	−22	32	5.05	L postcentral gyrus
		−52	−4	6	4.86	L parietal operculum
		−56	2	0	4.83	L parietal operculum
		−52	0	2	4.71	L parietal operculum
		−48	−2	8	4.69	L parietal operculum
		−50	−22	36	4.60	L postcentral gyrus
		−34	−20	10	4.21	L middle insula
		−34	−16	4	4.06	L middle insula
<0.001	2141	38	2	14	7.51	R middle insula
		38	−2	10	7.51	R middle insula
		56	−14	18	7.37	R parietal operculum
		58	−16	14	7.13	R parietal operculum
		44	−20	22	6.71	R parietal operculum
		46	−4	18	6.40	R parietal operculum
		38	−32	24	5.56	R posterior insula
		66	−10	8	5.54	R parietal operculum
		42	−32	26	5.43	R parietal operculum
		32	−16	22	5.26	R middle insula
		36	−8	20	5.23	R middle insula
		58	4	8	5.02	R parietal operculum
		34	−26	24	4.38	R posterior insula
		62	−2	6	4.34	R parietal operculum
0.005	415	10	12	−16	4.95	R orbitofrontal cortex
		−8	20	−18	4.85	L orbitofrontal cortex
		−16	24	−4	4.33	L caudate
		6	26	−16	4.27	R orbitofrontal cortex
		−10	22	2	4.21	L caudate
		0	26	−16	4.13	orbitofrontal cortex
		8	22	−16	4.12	R orbitofrontal cortex
		−14	20	−6	4.10	L caudate
		0	30	−14	4.09	orbitofrontal cortex
		−6	32	−12	4.03	L orbitofrontal cortex
		−18	36	−6	4.02	L orbitofrontal cortex
		−10	20	10	3.90	L caudate
*NE* > *PE*						
<0.001	3098	12	16	64	9.09	R medial superior frontal gyrus
		12	20	60	8.90	R medial superior frontal gyrus
		−12	8	66	7.52	L medial superior frontal gyrus
		10	6	68	7.36	R medial superior frontal gyrus
		−8	20	46	6.31	L dorsal anterior cingulate cortex
		−4	20	58	5.77	L dorsal anterior cingulate cortex
		6	30	50	5.58	R dorsal anterior cingulate cortex
		−4	38	42	5.13	L medial superior frontal gyrus
		8	18	40	4.84	R dorsal anterior cingulate cortex
		−6	26	36	4.76	L dorsal anterior cingulate cortex
		10	54	30	4.68	R medial superior frontal gyrus
		22	−2	70	4.36	R superior frontal gyrus
		20	54	28	3.70	R superior frontal gyrus
		24	54	24	3.59	R superior frontal gyrus
<0.001	2496	42	22	−16	8.54	R inferior frontal gyrus
		48	24	−8	8.16	R inferior frontal gyrus
		44	18	22	7.82	R inferior frontal gyrus
		46	22	4	6.82	R inferior frontal gyrus
		46	22	42	6.66	R middle frontal gyrus
		48	20	38	6.54	R inferior frontal gyrus
		42	22	−22	6.40	R temporal pole
		56	26	20	6.23	R inferior frontal gyrus
		50	36	−4	5.37	R inferior frontal gyrus
		36	8	42	5.15	R middle frontal gyrus
		38	6	46	5.09	R middle frontal gyrus
		42	8	52	5.04	R middle frontal gyrus
		42	44	−14	4.83	R inferior frontal gyrus
		42	40	−12	4.75	R inferior frontal gyrus
		34	10	36	4.75	R middle frontal gyrus
		50	16	−26	4.66	R temporal pole
<0.001	1082	54	−14	−12	7.24	R superior temporal sulcus
		50	−26	−8	7.06	R superior temporal sulcus
		54	−34	−2	5.60	R superior temporal sulcus
<0.001	2215	−46	20	8	6.78	L inferior frontal gyrus
		−44	18	−4	6.15	L anterior insula
		−34	20	−12	6.04	L anterior insula
		−54	26	16	5.67	L inferior frontal gyrus
		−56	24	12	5.48	L inferior frontal gyrus
		−46	6	42	5.06	L precentral gyrus
		−50	12	32	4.57	L precentral gyrus
		−44	50	−6	4.57	L middle frontal gyrus
		−44	50	−12	4.48	L middle frontal gyrus
		−48	46	0	4.34	L inferior frontal gyrus
		−46	10	34	4.31	L inferior frontal gyrus
		−44	22	24	3.73	L inferior frontal gyrus
		−48	36	24	3.68	L inferior frontal gyrus
		−42	8	24	3.58	L inferior frontal gyrus
0.021	296	64	−46	30	5.70	R temporoparietal junction
		62	−48	26	5.13	R temporoparietal junction
		50	−52	32	4.64	R temporoparietal junction
		52	−54	36	4.46	R temporoparietal junction

Activation was thresholded at an uncorrected *p* < 0.001 at the voxel level and a family-wise error (FWE) corrected *p* < 0.05 at the cluster level. Labels were determined by using mean normalized T1 images of the 26 participants and WFU PickAtlas. We did not include a significant cluster of PE > NE (located at (44, −16, 56)) in the table, which showed negative beta value in PE condition. Furthermore, we did not include peak information located at white matter. R = right; L = left.

### fMRI Results: Evaluation Sensitivity–Related Activation

**Figure 5:**
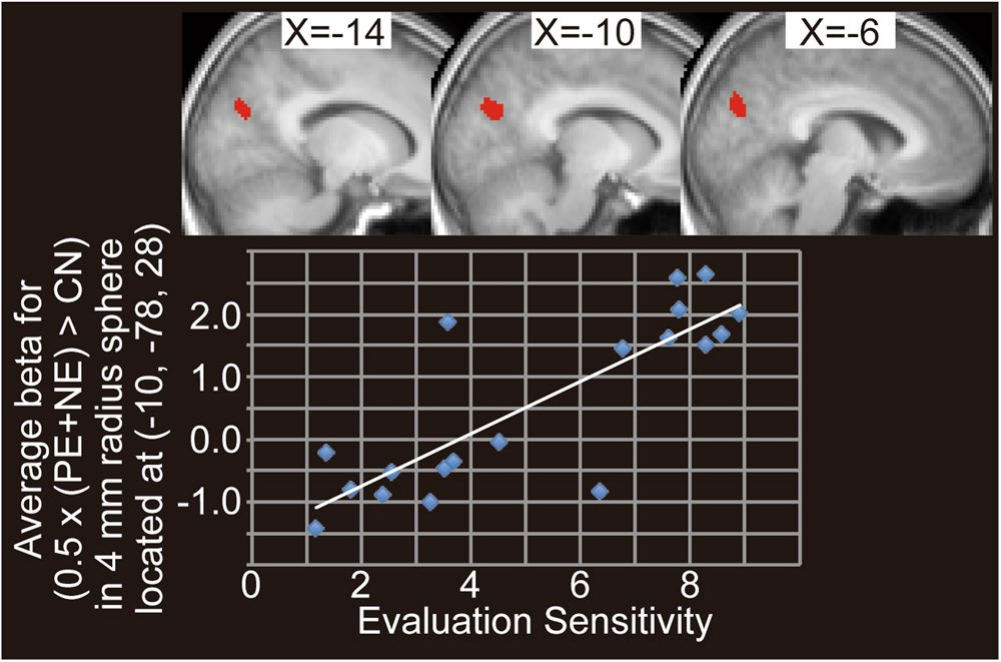
Precuneus activation related to evaluation effects positively correlated with evaluation sensitivity. Cluster of the left precuneus for (average of positive evaluation [PE] and negative evaluation [NE] > control [CN]) was overlaid onto the mean normalized T1 image of 19 participants. Scatter diagram of evaluation sensitivity and average beta-values of (0.5 × (PE + NE) > CN) in spheres of 4-mm radius located at the peak (−10, −78, 28) of the left precuneus cluster are shown at lower side. Activation threshold was set at an uncorrected *p* < 0.001 at the voxel level and a family-wise error (FWE) corrected *p* < 0.05 at the cluster level.

The left precuneus cluster exhibited a positive correlation with evaluation sensitivity in 0.5 × (PE + NE) > CN, i.e., the average of the two evaluation conditions (PE and NE) in comparison with control condition (CN) (Fig. 5 and Table 2). On the other hand, we did not observe any significant cluster exhibiting significant correlation with expected pleasantness.

**Table 2:** Evaluation sensitivity–related brain activation for evaluation effects.

Cluster *p* (FWE)	Cluster size	x	y	z	*t* value	Label
0.042	213	−10	−78	28	6.38	L precuneus

Average of positive evaluation (PE) and negative evaluation (NE) in comparison to control (CN) revealed significant clusters in the left precuneus/occipital cortex (OC), which exhibited a positive correlation with evaluation sensitivity. Label was determined by using mean normalized T1 images of the 19 participants and WFU PickAtlas. Activation was thresholded at an uncorrected *p* < 0.001 at the voxel level and a family-wise error (FWE) corrected *p* < 0.05 at the cluster level. L = left.

### Results of Functional Connectivity Analysis: Functional Connectivity with Evaluation Sensitivity-Related Regions

**Figure 6:**
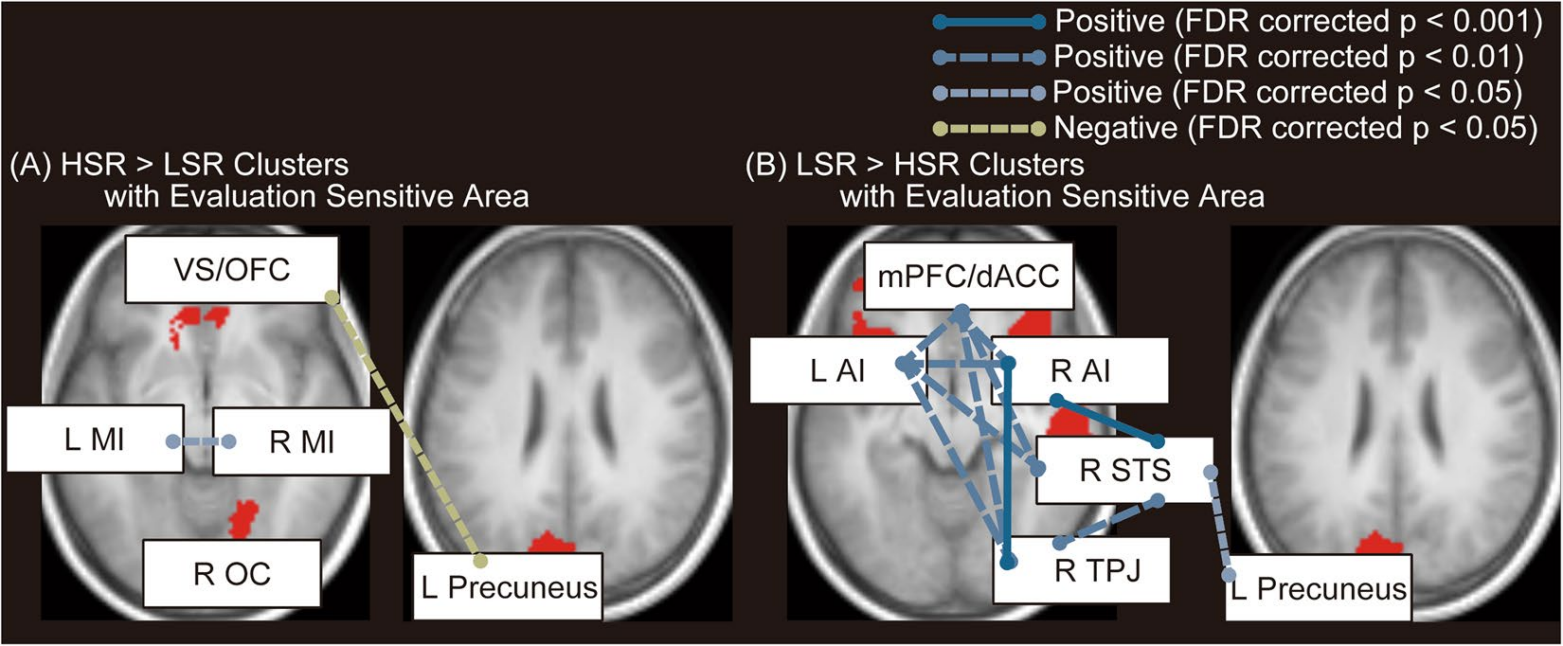
Significant functional connectivity within evaluation representation and evaluation sensitivity-related areas during resting state. (**A**) Functional connectivity within positive evaluation–related regions and evaluation sensitivity–related area (left precuneus), which exhibited significant correlation with evaluation sensitivity, is shown. (**B**) Functional connectivity within negative evaluation–related regions and evaluation sensitivity–related area (left precuneus), which exhibited significant correlation with evaluation sensitivity, is shown. Blue and green lines indicate positive and negative significant correlation, respectively. Threshold was false discovery rate (FDR) corrected *p* < 0.05. VS = ventral striatum; OFC = orbitofrontal cortex; R = right; L = left; MI = middle insula; OC = occipital cortex; mPFC = medial prefrontal cortex; dACC = dorsal anterior cingulate cortex; AI = anterior insula; STS = superior temporal sulcus; TPJ = temporoparietal junction.

In terms of functional connectivity within the left precuneus and positive evaluation–related regions, ROI-to-ROI analysis revealed that functional connectivity between the left precuneus and OFC/VS was significantly negatively correlated with evaluation sensitivity. Furthermore, functional connectivity between the bilateral middle insula exhibited a significant positive correlation with evaluation sensitivity (Fig. 6A).

In terms of functional connectivity within the left precuneus and negative evaluation–related regions, ROI-to-ROI analysis revealed that functional connectivity between the right STS and left precuneus was significantly positively correlated with evaluation sensitivity. Furthermore, functional connectivity within negative evaluation regions was significantly positively correlated with evaluation sensitivity (Fig. 6B).

## Discussion

### Change in State Self-esteem

In this study, we required participants to rate the pleasantness of evaluations from others and their own self-esteem (“your value”) in alternate trials. We considered the pleasantness rating as the subjective interpretation of the other’s evaluation. Because state self-esteem is a momentary feeling of self-worth, which consists of performance, social, and appearance self-esteem^8^, we anticipated that the pleasantness should contribute to the change in the state self-esteem to varying degrees across individuals. As expected, a majority of participants (75%) exhibited a significant positive correlation between pleasantness and change in state self-esteem, i.e., they were sensitive to others’ evaluation regarding their state self-esteem. On the other hand, such positive correlation was not observed in the rest of the participants. Furthermore, the evaluation sensitivity (i.e., the change in state self-esteem per unit change in pleasantness) varied across the participants (Fig. 5). The variability in evaluation sensitivity across participants can be explained by two factors. First, self-esteem is composed of a temporary component, state self-esteem, and a more stable component, trait self-esteem, which may vary across subjects. Second, state self-esteem is influenced not only by others’ evaluation as reflected by their use of a socially desirable adjective (such as “trustworthy,” “sincere,” etc.) used in this study, but also by adjectives related to performance and appearance. Some participants might not have paid attention to social self-esteem in comparison with the other two elements, neither of which were included in the evaluation words used in this study. Therefore, our finding of a positive correlation between pleasantness and change in state self-esteem suggested that state self-esteem is influenced by others’ evaluations, via the interpretation of these evaluations (perceived pleasantness), with varying sensitivity across participants.

### Activation Evoked by Negative and Positive Evaluation by Others

As expected, we observed significant activation in the dACC and AI, in addition to the right STS, in response to negative evaluation, consistent with the results of a previous study^11^. The right STS plays key roles in processing social information^21–23^ related to understanding others’ intentions^24^. Such understanding is necessary for processing one’s reputation (e.g., inferring reasons for reputation reception), leading to updating of state self-esteem. The dACC and AI constitute affective components^25^ of the pain matrix^26–28^. In addition to physical pain, both of these regions process psychological pain, including social exclusion^29,30^ or empathy for other’s psychological or physical pain^25,31–33^. Because activations related to experiencing physical pain and perceiving others’ pain often overlap^25,34,35^, dACC and AI are involved a common function related to processing of psychological and physiological pain stimuli^36^. Furthermore, highly narcissistic men, in whom others’ evaluation can result in fluctuation of self-esteem, exhibited dACC activation upon viewing their own faces^37^. Thus, dACC activation reflects negative emotion for a wide range of social situations. In this study, based on the pleasantness rating results, receipt of negative evaluation from others caused aversive feelings. In this sense, activation of dACC and AI represents aversive processing, which is common to multiple forms of pain processing^38^.

In response to positive evaluation, we observed activation of VS as well as OFC. The striatum is the primary input structure of the basal ganglia^39^, and one of its major roles is reward processing^40^. The VS represents the common currency of reward^17^, including abstract rewards (e.g., being actively listened to^41^ or receiving praise from others^17^) and monetary gain^17,42,43^. Also, OFC may integrate reward value across different stimuli or stimulus dimensions^44^, and also represents social reward^45^. We observed that in contrast to receipt of negative evaluation, receipt of positive evaluation evoked pleasant feelings, indicating that OFC/VS activation is evoked by receipt of a social reward, i.e., positive evaluation. Taken together, activation of the affective pain matrix in response to negative evaluation, and of the reward system in response to positive evaluation, suggested that our experimental conditions for negative and positive evaluation were properly defined.

### Activation Related to Evaluation Sensitivity

Precuneus activity evoked by reputation rating was positively correlated with evaluation sensitivity. Because evaluation sensitivity indicates how sensitively an individual’s state self-esteem is influenced by others’ appraisal, the correlation suggests that the precuneus is involved in integration of the subjective interpretation of others’ appraisal with state self-esteem.

The precuneus and mPFC are closely connected, forming part of the network involved in mentalizing, and are involved in self-reflection^46–48^. Previous studies showed that mPFC activation^11,49^ or mPFC connectivity with striatum^7^ represents trait and state self-esteem. Although the mPFC is involved in abstract representation of the self^50^, the precuneus is associated with long-term memory of self-related information, i.e., episodic memory^51–53^. Both trait and state self-esteem rely heavily on abstract representation of the self. On the other hand, updating state self-esteem is based on former state self-esteem in addition to representation of one’s reputation following receipt of evaluation from others (a type of self-related information). Because former state self-esteem is based on the history of previously received evaluations from others, long-term memory of self-related information is necessary for change in state self-esteem. Previous studies showed that retrieving and constructing information related to the self (fundamental information of state self-esteem) is represented in the precuneus^19,20^. In this sense, present study advances our understanding of self-related functions in the precuneus. Specifically, the precuneus is involved in updating state self-esteem by transforming others’ evaluation of oneself into state self-esteem, thereby functioning as a gateway into the mentalizing system for subjective evaluation regarding others’ appraisals.

### Functional Integration Between Negative and Positive Evaluation Representation and Change in Self-Esteem

ROI-to-ROI analysis of resting-state fMRI revealed significant functional connectivity between the evaluation sensitivity-related area (i.e., the left precuneus) and the right STS. This functional connectivity was significantly positively correlated with evaluation sensitivity. Furthermore, the right STS exhibited significant functional connectivity with other areas involved in representation of negative evaluation (i.e., mPFC/dACC, IFG/AI, and right TPJ). In this sense, the right STS acts as an information hub between representation of negative evaluation and transformation of self-esteem. Such function is necessary for processing one’s reputation (e.g., inferring reasons for reputation reception), leading to updating of state self-esteem.

In contrast to negative evaluations, we observed a significant negative correlation between evaluation sensitivity and functional connectivity between the left precuneus and OFC/VS. This finding indicates that the higher the evaluation sensitivity, the lower the influence of the OFC/VS towards the precuneus. Because the OFC/VS is involved in positive evaluation, this suggests that evaluation sensitivity represented by the precuneus is biased towards negative evaluation. That is, the precuneus acts as a gateway that increases information exchange with regions involved in negative evaluation while decreasing exchange with those involved in positive evaluation. This is consistent with the hypothesized sociometer, which is postulated to alert the individual to the possibility of social exclusion^3^. This result is supported by a previous study in which brain activation of state self-esteem only for negative evaluation was detected by fMRI^11^. Furthermore, our results were also supported by a previous study showing that narcissism, in parallel with self-esteem fluctuation, is associated with weakened structural connectivity between self-related and reward areas^54^. Based on the results of this and the previous study, we conclude that precuneus acts as a gateway regarding functional segregation, especially for higher sensitivity to others’ evaluations, leading people with higher evaluation sensitivity to pay attention to others’ responses, especially in the context of negative interactions.

### Limitations

There were several limitations in the present study. (1) Because 25 percent of the participants did not exhibit a significant correlation between pleasantness and change in state self-esteem (transformation function), future studies are necessary to investigate the differences between the two groups (i.e., those that exhibited a significant correlation and those that did not). (2) In this study, resting-state fMRI was conducted after task-based fMRI. Therefore, our functional connectivity results regarding evaluation sensitivity might have been influenced by evaluation processing during task-based fMRI (i.e., some kind of order effect). Because task fMRI might increase detection power of brain activation related to evaluation sensitivity through invoking evaluation processing, this manipulation might be useful for investigating brain mechanisms related to evaluation sensitivity. To evaluate the possibility of an order effect, we are planning future studies to test whether we can modulate processing related to participants’ evaluation sensitivity.

## Conclusions

Changes in self-esteem based on self-image assessment play an essential role in maintaining well-being in human society. In this study, we found that significant activation of the precuneus covaried with evaluation sensitivity in the context of evaluation reception. In resting-state fMRI, the precuneus exhibited significant functional connectivity with the right STS, which was activated in response to negative evaluation. On the other hand, in the context of positive evaluation, elevated evaluation sensitivity might weaken the functional connectivity between the left precuneus and OFC/VS. Thus, changes in self-esteem are represented in the precuneus with interrelating to negative or positive evaluation activation. We conclude that the precuneus, as the part of the mentalizing system, serves as a gateway for translating the subjective evaluation of reputation into state self-esteem.

## Methods

### Participants

Twenty-eight participants (15 men, 13 women) took part in the experiment. The average age ± SEM of the participants was 20.64 ± 0.43 years (women, 19.92 ± 0.34 years; men, 21.19 ± 0.67 years). All participants had normal or corrected-to-normal visual acuity. All participants were right-handed according to the Edinburgh handedness inventory^55^. Participants received monetary compensation for their time. The protocol was approved by the ethical committee of the National Institute for Physiological Sciences, Okazaki, Japan. The experiments were undertaken in compliance with national legislation and the Code of Ethical Principles for Medical Research Involving Human Subjects of the World Medical Association (Declaration of Helsinki). All participants provided written informed consent.

### Visual Stimuli Presentation

Visual stimuli were presented using the Presentation software v. 16.4 (Neurobehavioral Systems, Inc.) implemented on a personal computer (dc7900; Hewlett-Packard, Ltd.). A liquid crystal display (LCD) projector (CP-SX12000; Hitachi, Ltd.) located outside and behind the scanner projected the stimuli through a waveguide to a translucent screen, which the participants viewed via a mirror placed in the MRI scanner. The spatial resolution of the projector was 1,024 × 768 pixels, with a 60-Hz refresh rate. The distance between the screen and the participant’s eyes was approximately 175 cm, and the visual angle was 13.8° (horizontal) × 10.4° (vertical). Responses were collected via an optical button box (Current Designs, Inc.).

### Task Design: Reputation Reception Experiment

The participants participated in 2-day sessions, as in previous studies^17,19^. The time between the first and second experimental sessions was 7 days.

On the first day, the participants took part separately in a self-introduction session, during which they initially completed a self-introduction sheet containing open-ended questions such as “What do you do in your free time?”, “What is your personality like?”, “What are your goals for the future?”, and “Please pick one problem that modern Japanese society faces and briefly state your opinion for tackling the issue”. After completing the self-introduction sheet, they were required to tell a self-introduction story, which was video-recorded. At the beginning of the session, they were told that the information provided would be viewed by eight people (two groups of four evaluators), who would then form an impression of the participant, as in previous studies^17,19^. At that time, we confirmed that the evaluators were strangers to the participants.

On the second day, we took photos of the participants’ faces, which were used for the following fMRI experiment. Then, the participants took part in the fMRI experiment, during which they were presented with the results of impression evaluations (adjectives), along with a photo of themselves, for 5 s (feedback trial). We instructed the participants to maintain a neutral facial expression when the photo was taken. The adjectives were presented in a predetermined order. We selected 60 adjectives from 84 items used in a previous study^17^, similar to a previous study^19^. The adjectives were selected based on the rating results of ten independent evaluators (six men) using a seven-point Likert scale (ranging from 7, ‘highly socially desirable,’ to 1, ‘not at all’). The mean ± SEM social desirability rating of the 30 positive evaluation words was 5.97 ± 0.06. On the other hand, the mean ± SEM social desirability rating of the 30 negative evaluation words was 2.99 ± 0.15. Twenty of the 60 items were presented twice, as large portion of duplicability made participants to think that evaluation words were commonly and independently selected by both groups of evaluators. These 20 items were selected pseudo-randomly, similar to previous studies^17,19^. All participants were told that they would rate the eight evaluators after the fMRI experiment, and that our aim was to investigate the neural mechanisms underlying formation of first impressions.

The fMRI experiment used an event-related design with four conditions: feedback, button-press, self-esteem, and rest (Fig. 1). During the feedback trial, the participants were required to evaluate the perceived pleasantness of the items shown (adjectives with photos) on a seven-point scale (ranging from 7, ‘very much,’ to 1, ‘not at all’) using the right index and middle fingers and thumb (Fig. 1). Participants rated pleasantness by pressing buttons after a circle was shown in a random position on the scale. Pushing the right index finger moved the circle to the next position to the left. On the other hand, pushing the right middle finger moved the circle to the next position to the right. Participants finalized the rating using the right thumb. During the button-press trial, as in the feedback trial, the participants were asked to press a button with their right index and middle fingers and thumb. Furthermore, the participants were required to move the selected circle far right or far left. Five seconds after stimulus onset, the next stimulus was presented. In each fMRI run, the feedback trials were repeated 40 times, and the button-press trials were repeated 20 times. In addition, a fixation rest trial was repeated 20 times in each run. The self-esteem trial was presented at the first trial of each run and immediately after each feedback trial for 5 s. In the self-esteem trial, participants were required to rate their state self-esteem at that time using a visual analog scale (VAS). They used the right index and middle fingers and thumb to operate VAS to report state self-esteem (ranging from 0 to 100: 0, ‘very negative’; 100, ‘very positive’). Pushing the right index finger decreased the value of VAS, whereas, pushing the right middle finger increased the value of VAS. The duration of pushing time determined the magnitude of the value change. Participants finalized the rating using the right thumb. Prior to the fMRI measurements, we instructed the participants to rate their state self-esteem based on feedback reception from ostensible evaluators, as in a previous study^11^. Thus, the self-esteem trials were repeated 41 times in each fMRI run. There were two runs in each session. The trial sequence for each run was predetermined and counterbalanced across subjects. Prior to the fMRI experiment, the subjects took part in a 2-min practice session, in which the adjectives were different from those used in the fMRI experiment. The total duration of the run was 10 min 45 s.

### Resting-state fMRI

After the reputation reception experiment, we measured brain activation of the same 28 participants in the resting state with eyes open; the total duration of this phase of the measurement was 5 min 30 s. Before and after the resting-state fMRI measurements, the participants were required to rate themselves on the Stanford sleepiness scale^56^, a subjective measure of sleepiness ranging from 1 to 7: 1 corresponds to most alert, and 7 to most sleepy.

### fMRI Data Acquisition

A 3T scanner (Verio; Siemens, Ltd., Erlangen) was used for the fMRI study. Each subject’s head was immobilized within a 32-element phased-array head coil. fMRI was performed using an multiband echo planar imaging (EPI) gradient-echo sequence (echo time [TE] = 35 ms; repetition time [TR] = 1,000 ms; field of view [FOV] = 192 × 192 mm^2^; flip angle = 65°; matrix size = 96 × 96; 60 slices; slice thickness = 2 mm + 0.5 mm gap; total number of volumes = 645 for feedback experiments and 330 for resting)^57^. A whole-brain, high-resolution, T1-weighted anatomical MR image using magnetization-prepared rapid acquisition gradient echo (MP-RAGE) was also acquired for each subject (TE = 2.97 ms; TR = 1,800 ms; FOV = 256 × 256 mm^2^; flip angle = 9°; matrix size = 256 × 256 pixels; and slice thickness = 1 mm).

### Behavioral Data Analysis

The purpose of this experiment was to examine the neural substrates of change in self-esteem based on self-image evaluation. Accordingly, we focused our analysis on participants whose state self-esteem changed in response to the reputation. For this purpose, we conducted a simple regression analysis between the pleasantness rating and change in self-esteem rating (independent variable, pleasantness rating; dependent variable, change in self-esteem rating). Change in self-esteem was calculated by subtracting the prior value of state self-esteem from the current value of state self-esteem.

For subsequent fMRI analysis, we selected participants who exhibited a significant correlation between pleasantness rating and change in self-esteem rating. For each of the participants, using the least-squares method, we graphed the change in self-esteem vs. pleasantness (change in self-esteem = pleasantness × *a* + *b*), interpreting *a* (slope) as evaluation sensitivity and −1 × x-intercept (the value of pleasantness resulting in zero change to self-esteem) as expected pleasantness. Because humans evaluate reward value using a temporal difference function (relative reward value)^58^, perception of relative pleasantness (difference relative to expected pleasantness) might be essential for transforming state self-esteem based on reputation evaluation. In this sense, expected pleasantness might be another key factor in the transformation of perceived pleasantness to change in state self-esteem.

### fMRI Data Analysis: Brain Activation Evoked by Evaluation Reception

We used SPM8 revision 5236 (The Wellcome Trust Centre for Neuroimaging; http://www.fil.ion.ucl.ac.uk/spm) implemented in MATLAB 2013a (MathWorks, Inc., Massachusetts) to analyze the functional images. The first ten volumes of each fMRI run were discarded because the MRI signal was unsteady. On the remaining volumes, we performed motion correction and normalization to the Montreal Neurological Institute (MNI) template. Then, the anatomically normalized EPI images were resampled to a voxel size of 2 mm × 2 mm × 2 mm and spatially smoothed using a Gaussian kernel of 8 mm (full width at half maximum, FWHM). After the realignment processes, we checked the head movement parameters. The task-related activation was evaluated statistically on a voxel-by-voxel basis using a general linear model at the individual level to generate contrast images, which then were incorporated into random-effects analysis at the group level^59^. In the fMRI data analysis, we excluded data from two participants who exhibited excessive body movements (over 2-mm spike-like movements during runs); consequently, data from 26 participants were analyzed.

Evaluation conditions were classified into three types based on the results of pleasantness ratings: PE, intermediate evaluation, and NE. Because pleasantness for received evaluation was dependent on each participant, we defined positive and negative evaluation conditions using each participant’s median pleasantness scores. Positive evaluation events were defined as evaluations for which the pleasantness score was higher than the median of the participant’s pleasantness scores. On the other hand, negative evaluation events were defined as evaluations for which the pleasantness score was lower than the median pleasantness score. Finally, intermediate evaluation events were defined as evaluations in which the pleasantness score was equal to the median. Based on the definition regarding intermediate evaluation (the median of individual pleasantness ratings), the number of intermediate evaluation trials was small compared to the number of PE and NE conditions. Means ± SEM trials were as follows: PE = 31.81 ± 1.75 trials; intermediate = 12.62 ± 2.10 trials; NE = 34.85 ± 0.67 trials. Accordingly, we did not use intermediate evaluation in the second-level analysis. Thus, in the design matrix of the individual-level analysis, we defined eight types of regressors (PE, intermediate evaluation, NE, CN, self-esteem, and three regressors for trials with misses [for each evaluation condition, CN, and self-esteem]) and six regressors, each representing motion parameters. In CN, participants were required only to press the button (button press trial). The duration of each trial for the task regressors (PE, intermediate evaluation, NE, CN, self-esteem, and miss) was 5 s. The time series for each voxel was high-pass filtered at 1/128 Hz. Assuming a first-order autoregressive model, the serial autocorrelation was estimated from the pooled active voxels with the restricted maximum likelihood (ReML) procedure, and used to whiten the data^60^. To investigate the neurological difference between receipt of positive and negative evaluations, we compared brain activation between PE and NE conditions in the individual-level analysis. We then conducted group analyses using the one-sample *t*-test by applying an exclusive mask, which revealed significant activation during CN (threshold was at an uncorrected *p* < 0.001 at the voxel level, and family-wise error (FWE) corrected *p* < 0.05 at the cluster level) for excluding button press-related activation during CN. Activation was thresholded at an uncorrected *p* < 0.001 at the voxel level and an FWE corrected *p* < 0.05 at the cluster level.

### fMRI Data Analysis: Brain Activation Covaried with Evaluation Sensitivity

In this fMRI data analysis, we excluded data from two participants who exhibited excessive body movements (mentioned above), as well as participants who did not exhibit significant positive correlation between change in self-esteem and pleasantness score.

In the behavioral analysis, we observed a significant positive correlation between pleasantness score and change in self-esteem rating in 21 out of 28 participants (described below). Based on this behavioral result, we investigated the neural correlates underlying the transformation function from pleasantness of receiving an evaluation to change in self-esteem. In the individual-level analysis, we defined contrast of evaluation-related effects (0.5 × (PE + NE) > CN) of 19 participants (excluding two participants exhibiting excessive body movement and seven participants who did not exhibit the aforementioned correlation, i.e., a transformation function) based on preprocessed data described above. Because the transformation function varied from individual to individual, we conducted group-level analysis by applying the two parameters (evaluation sensitivity and expected pleasantness) of the transformation function as covariates for investigating the neural correlates of the personal transformation function. We did not detect a significant correlation between these two parameters (R = 0.16, *p* = 0.685). Using this model, we evaluated evaluation sensitivity effects, which exhibited a positive correlation with evaluation sensitivity, by applying an exclusive mask. The exclusive mask consisted of significant clusters during CN (threshold was at an uncorrected *p* < 0.001 at the voxel level and a FWE corrected *p* < 0.05 at the cluster level). The aim of setting this exclusive mask was to exclude button press–related effects. Activation was thresholded at an uncorrected *p* < 0.001 at the voxel level and FWE corrected *p* < 0.05 at the cluster level.

### Functional Connectivity Analysis

Next, we conducted functional connectivity analysis of resting-state fMRI data to investigate the functional relationship within the transformation function, i.e., functional connectivity within negative or positive evaluation representation (self-image assessment) regions and evaluation-sensitive regions. In this analysis, we preprocessed resting-state fMRI data via the aforementioned steps using SPM8. With the preprocessed data, we performed functional connectivity analysis using the CONN toolbox (version 15.h). In the individual-level analysis, we defined the whole run as a rest trial for 19 participants. Because these 19 participants did not give a rating of 6 (“felt sleepy, would have preferred to lie down, woozy”) or 7 (“could not stay awake, sleep onset was imminent”) on the Stanford sleepiness scale, we assumed that they did not fall asleep during resting-state fMRI measurements. In the group-level analysis, we set evaluation sensitivity and expected pleasantness as covariates, and negative evaluation regions (NE > PE) or positive evaluation regions (PE > NE), in addition to evaluation sensitivity regions, as regions of interest (ROIs). Using these ROIs, we performed ROI-to-ROI analysis. Each ROI was a 4-mm radius sphere located at a peak of a significant cluster. ROI-to-ROI functional connectivity was thresholded at a false discovery rate (FDR) corrected *p* < 0.05.

### Data availability

The datasets generated during the current study are available from the corresponding author on reasonable request.
